# Ectopic Fgf signaling induces the intercalary response in developing chicken limb buds

**DOI:** 10.1186/s40851-018-0090-2

**Published:** 2018-04-19

**Authors:** Aki Makanae, Akira Satoh

**Affiliations:** Research Core for Interdisciplinary Sciences (RCIS), Okayama University, 3-1-1, Tsushimanaka, Kita-ku, Okayama, 700-8530 Japan

**Keywords:** Limb development, *HoxA11*, Intercalation, Limb regeneration, Fgf signaling, Chick, Axolotl

## Abstract

**Background:**

Intercalary pattern formation is an important regulatory step in amphibian limb regeneration. Amphibian limb regeneration is composed of multiple steps, including wounding, blastema formation, and intercalary pattern formation. Attempts have been made to transfer insights from regeneration-competent animals to regeneration-incompetent animalsat each step in the regeneration process. In the present study, we focused on the intercalary mechanism in chick limb buds. In amphibian limb regeneration, a proximodistal axis is organized as soon as a regenerating blastema is induced. Intermediate structures are subsequently induced (intercalated) between the established proximal and distal identities. Intercalary tissues are derived from proximal tissues. Fgf signaling mediates the intercalary response in amphibian limb regeneration.

**Results:**

We attempted to transfer insights into intercalary regeneration from amphibian models to the chick limb bud. The zeugopodial part was dissected out, and the distal and proximal parts were conjunct at st. 24. Delivering ectopic Fgf2 + Fgf8 between the distal and proximal parts resulted in induction of zeugopodial elements. Examination of HoxA11 expression, apoptosis, and cell proliferation provides insights to compare with those in the intercalary mechanism of amphibian limb regeneration. Furthermore, the cellular contribution was investigated in both the chicken intercalary response and that of axolotl limb regeneration.

**Conclusions:**

We developed new insights into cellular contribution in amphibian intercalary regeneration, and found consistency between axolotl and chicken intercalary responses. Our findings demonstrate that the same principal of limb regeneration functions between regeneration-competent and -incompetent animals. In this context, we propose the feasibility of the induction of the regeneration response in amniotes.

**Electronic supplementary material:**

The online version of this article (10.1186/s40851-018-0090-2) contains supplementary material, which is available to authorized users.

## Background

The intercalary mechanism is considered a primary principle of regeneration in regenerative animals [1–5]. Intercalary pattern formation occurs following blastema formation. Blastema formation is considered to be the primary issue, since regeneration-incompetent animals, such as amniotes, do not form a blastema after damage. In blastema formation, the formation of an amputation plane is usually the start of blastema formation. The surrounding epidermis starts migrating to cover the exposed surface, forming wound epidermis/epithelium (WE). Undifferentiated cells are accumulated beneath the WE, forming a regeneration blastema. Regeneration blastemas are thought to have a similar structure as developing limb buds. A similar or the same limb patterning mechanism as that for a developing limb bud appears to take place in order to remake a limb. The patterning mechanism in regeneration has been considered the intercalary pattern-forming mechanism. A regeneration blastema and an amputated plane initially re-establish a distal and a proximal positional identity, respectively [6–8]. Intermediate structures are then induced between the distal and the proximal tissues. This induction of an intermediate region by an established distal and an amputated stump is called “intercalation.” These intercalary pattern-forming mechanisms in regeneration are conserved among species [1, 4].

Transferring the insights gained from regenerative animals to non-regenerative animals has been attempted. The first step, blastema formation, has been a significant challenge thus far, however as regeneration-incompetent animals cannot form a blastema. For example, mammalians cannot grow a blastema after limb amputation. Only the very distal tip retains regeneration potency. It is known that mice and even humans can regrow digit tips [9]. Generally, proximal amputation results in no regeneration response (no growth) in mice digits [10, 11]. The application of Bmp proteins, however, can successfully induce the regeneration response [12]. However, the re-induction of a developmental field in a mature body has been a challenge in mice. The inability to induce a blastema after amputation can be also observed from the embryonic period in regeneration-incompetent animals. When a developing chick limb bud is amputated, the amputated limb bud cannot regenerate the damaged region, and a truncated limb results [13, 14]. Even if an amputation is performed in the quite early stages, in which no skeletal elements are specified in a developing limb bud, no regeneration responses are observed. Interestingly, ectopic Fgf expression onto an amputated surface in the early stages can induce a distal structure [13, 15, 16]. Activation Fgf signaling induces regeneration responses, which appears to be a mechanism conserved from amphibians to chick embryos [17–19]. This induction of regeneration responses by exogenous Fgf signaling, however, can take place within the quite early developmental stages. In later stages, including in adult chicks, limb regeneration responses are no longer inducible by ectopic Fgfs [16]. Therefore, blastema induction remains a primary issue in regeneration-incompetent animals. This has prevented further study beyond blastema formation in regeneration-incompetent animals.

Although the induction of a blastema continues to be a challenge, the use of chicken embryos allows us to investigate the latter phase, i.e. the intercalary pattern formation, in amniotes. Chicken embryos are easy to access if the eggshell is removed. Once an embryo is exposed, the limb buds are easy to manipulate. It is of note that chick embryos cannot regenerate amputated limb buds unless ectopic Fgf is applied to the amputated limb bud in the quite early stages [13, 15, 16]. Due to the easy accessibility of a chick embryo, it is possible to create a “pseudo-blastema” on an amputated limb bud. As mentioned above, a blastema is similar in structure to the distal part of a developing limb bud. When a chick limb bud is amputated, a distal part is always dissected out. In this study, we regarded an abscised distal part as a pseudo-blastema and placed it onto an amputated stump of a chick limb bud after dissecting out the expected zeugopodial region. This experimental design allows study of the intercalary processes that occur after blastema induction.

The present study focuses on the induction of the intercalary response in chick limb buds. Fgf signaling has been suggested to be involved in the process [7, 20–22]. In amphibians, when the intermediate structures were removed and a distal part (a hand part or a developing distal limb bud) was placed onto an amputated proximal structure, no intermediate regeneration could be observed. Ectopic Fgf application, however, resulted in the induction of intermediate structures in amphibians [21, 22]. This is thought to be regeneration of an intermediate structure. We aimed to investigate this intermediate regeneration in chick limb buds. Hamburger and Hamilton stage 24 (st. 24) chicken limb buds, in which most zeugopodial elements were already specified, were dissected into three pieces [23, 24]. The presumptive zeugopodial parts were removed, and the two pieces, the distal and proximal parts, were joined. We found that ectopic Fgf2 + 8 application resulted in induction of the zeugopodial elements. Cell lineage tracing provides comparative insights to amphibian limb regeneration. Our findings suggest that induction regeneration responses in the limb buds of chick embryos are possible if discontinuity of the distal and proximal positional values is created under the presence of active Fgf ability. In other words, the primary principal of limb regeneration, the intercalary response, can be induced in a regeneration-incompetent chick embryo.

## Methods

### Experimental manipulations

Chicken embryos were staged according to the methods of Hamburger and Hamilton [23]. Stage 24 limb buds were amputated at 250 μm and 800 μm from the distal tip with forceps. Distal fragments of the dissected tissues were grafted onto proximal stamps with Fgf2 + 8-soaked or PBS-soaked beads by tungsten needles. The beads were prepared following previously described methods [25]. To visualize the skeletal pattern, the embryos were incubated for 7 days after surgery and stained with Alcian blue.

Axolotls (*Ambystoma mexicanum*) with a nose-to-tail length of 8–12 cm were obtained from private breeders and housed in aerated water at 22 °C. Green fluorescent protein (GFP) transgenic axolotls were obtained from the Ambystoma Genetic Stock Center (AGSC). Hand grafting procedures followed the previous report [21].

### Sectioning and histological staining

Samples were fixed with 4% paraformaldehyde for 1 day at room temperature. If necessary, decalcification by 10% EDTA was performed for 1 day. Samples were embedded in O.C.T. compound (Sakura Finetek, Tokyo) following 30% sucrose/phosphate-buffered saline (PBS) treatment for approximately 12 h. Frozen sections of 14 μm thickness were prepared using a Leica CM1850. The sections were dried thoroughly under an air dryer and kept at − 80 °C until use.

Standard haematoxylin and eosin (HE) staining was used for histology. To visualize cartilage formation, Alcian blue staining was performed before HE staining. In brief, sections were washed in tap water several times to remove the O.C.T. compound. Then, Alcian blue (Wako, pH 2.0) solution was dropped on the section, and the slide was incubated for 5 min. The sections were washed twice with tap water, and then HE staining was performed. The stained sections were mounted using Softmount (Wako, Osaka). For whole-mount skeletal staining, we used the procedures reported by [21].

### In situ hybridization, immunofluorescence, and in situ apoptosis detection

The following genes were cloned by reverse transcription polymerase chain reaction (RT-PCR): *Pg-h*, *Meis2*, *Shox2*, *Hoxa-11*, and *Hoxa-13*. RNA probe templates were synthesized by PCR using M13 forward and reverse primers. Based on sequence data, the appropriate RNA polymerase was selected to synthesize antisense RNA probes. A labelled *Hoxa-11* probe was subjected to alkaline hydrolysis to obtain optimal signals. Whole-mount and section in situ hybridization for chick embryos was performed using standard methods. *Gdf5* and *type II collagen* probe for in situ hybridization for amphibians were described in the previous study [26, 27]. Immunofluorescence on sections was carried out on the basis of previous reports [28]. Chicken cell marker antibody (8F3, 1:200) and quail cell marker antibody (QCPN, 1:100) were obtained from the Developmental Studies Hybridoma Bank (DSHB). Anti-phospho-histone H3 antibody (1:500) was purchased from Cell Signaling Technology. Anti-GFP (1:500) was purchased from Clontech. Anti-mouse IgG Alexa 488 (1:500) and anti-rabbit IgG Alexa 488 (1:500) were purchased from Invitrogen. Nuclei were visualized by Hoechst 33,342 (Dojindo) staining. Images were captured using an Olympus BX51 system. All experiments were performed three times to confirm the results. Statistical analysis was performed by t-test (one-tail). Cell counting was performed in three independent samples.

Apoptotic cells were detected with an In Situ Apoptosis Detection Kit (TaKaRa Bio Inc.). Staining was performed according to the kit manual. Sections were washed in PBS and covered with permeabilization buffer for 5 min on ice. After the PBS wash, TdT labelling solution was dropped onto the slides before incubation at 37 °C for 90 min. The labelled slides were washed in PBS containing Hoechst 33,342 (Dojindo) and mounted coverslips. All experiments were performed three times to confirm the results. Statistical analysis was t-test. Cell counting was performed from three independent samples.

## Results

### Induction of intercalary responses in st. 24 chick limb buds

**Fig. 1:**
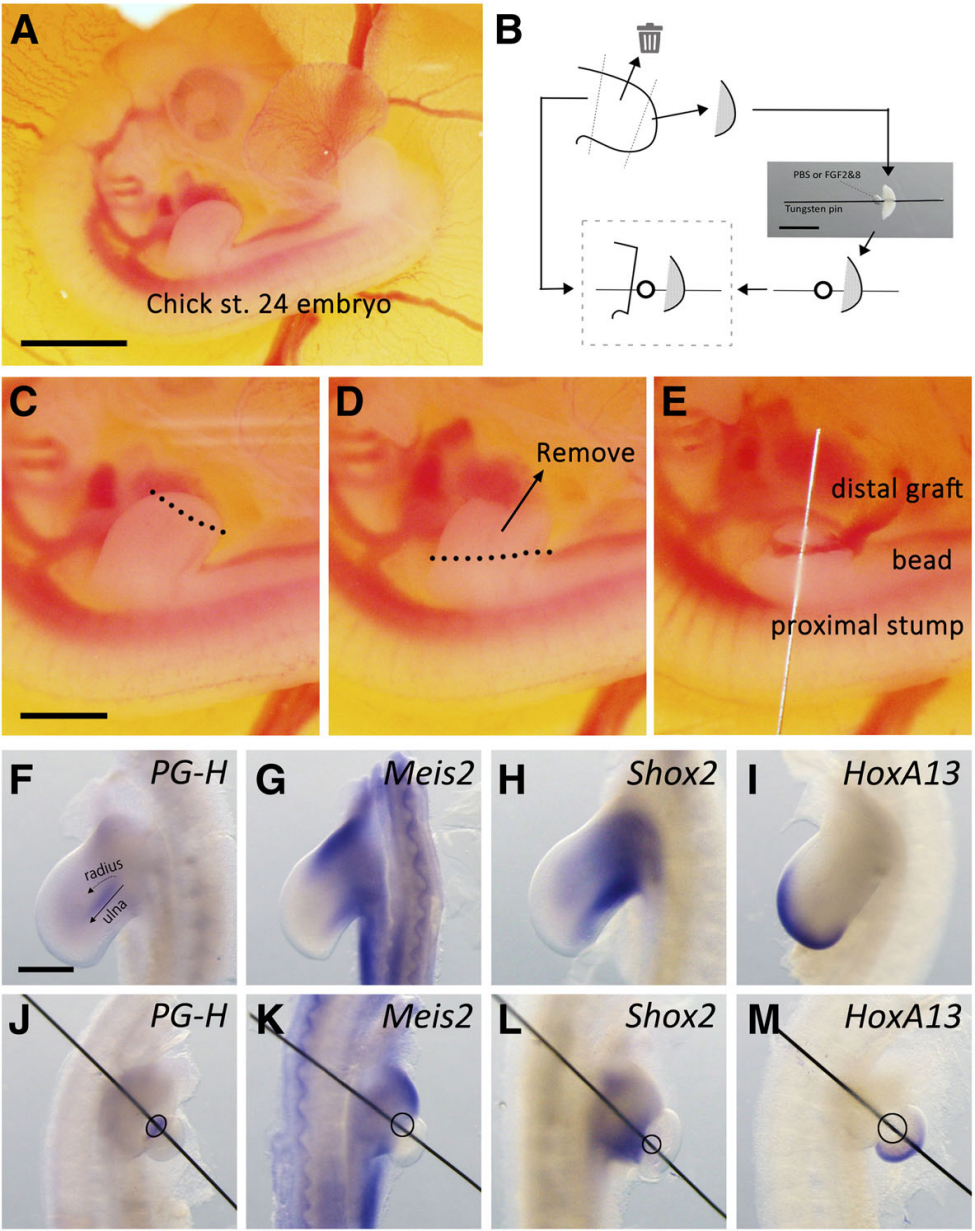
Summary of the experimental procedures and gene expression pattern. **a** Chicken embryo at stage 24, which was used for the surgery in the present study. **b**–**e** Summary of surgical procedures. **b** The schematic diagram of the surgical procedure. The distal one-quarter was isolated and stabbed with a gelatin bead. **c** The distal one-quarter of the limb bud was dissected out and the isolated part was used for the graft. **d** The next two quarters, the intermediate part, was removed. **e** The remaining one-quarter of the stump was stuck with the distal graft and the bead. **f**–**m** Gene expression pattern in non-amputated limb buds (**f**–**i**) and operated limb buds (**j**–**m**). *Pg-h* (*Aggrecan*) expression (**f**, **j**), *Meis2* expression (**g**, **k**), *Shox2* expression (**h**, **l**), and *HoxA13* expression (**i**, **m**). The circles indicate the position of the grafted bead (J–M). The scale bars in **a**, **b**, **c**, and F are 2, 1, 1, and 0.5 mm, respectively

The experimental design is illustrated in Fig. 1a–e. The experimental procedures are summarized in Fig. 1b. St. 24 chick embryos were used. Zeugopodial cartilaginous elements (radius/ulna) were targeted and dissected out, as shown in Fig. 1c–e. The distal part was placed onto the amputated stump, and an Fgf2 + 8-soaked or phosphate-buffered saline (PBS)-soaked bead was settled between the distal part and the proximal part (Fig. 1e). This surgery joined the distal part to the amputated stump, which is similar to when an early blastema forms on an amputated stylopod, since an early blastema possesses a distal identity. For the purpose of prospecting the st. 24 limb bud, in situ hybridization was performed (Fig. 1f–m). Limb buds at st. 24 already specified the zeugopodial elements (Fig. 1f). Cartilage-specific proteoglycan (*Pg-h*) expression visualizes the ulna elements although the radius continued to have no distinguishable PG-H expression ([29]; Fig. 1f). Stylopod cartilage is usually determined at this stage. *Pg-h* expression, however, could not be detected by whole-mount in situ hybridization, due to the thickness of the limb bud (a technical reason). The grafting procedure eliminated the zeugopodial elements. Therefore, the *Pg-h* expression domain in the zeugopod disappeared completely (Fig. 1j). *Meis2* and *Shox2* are the proximal marker genes, which mainly express in a stylopodium region ([30, 31]; Fig. 1g, h). These gene expressions could not be detected in the distal graft, indicating that there was no proximal cell contamination in the distal graft (Fig. 1k, l). *HoxA13* is an autopod marker gene (Fig. 1i). *HoxA13* was expressed in the grafted distal part but not in the proximal stump at all (Fig. 1m). These results support our choice of experimental design.

**Fig. 2:**
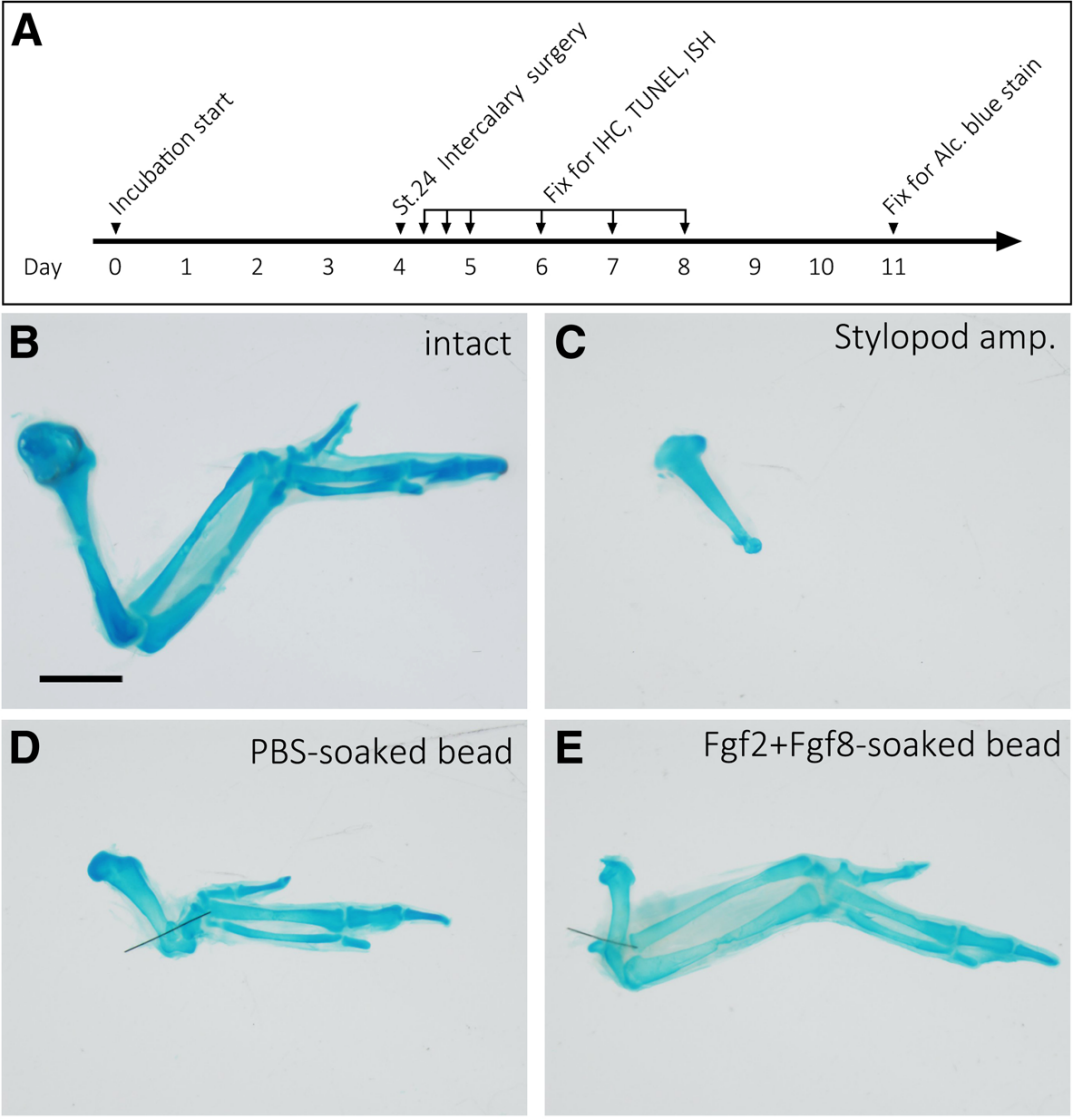
The skeletal phenotypes. **a** Time schedule of the experiment. **b** The intact chicken wing. **c** The skeletal pattern derived from the amputated limb bud three-quarters from the distal tip at st. 24. **d** The skeletal pattern of the limb bud from which the middle part was dissected out. An intercalary induced structure could be observed. **e** An Fgf2 + Fgf8-soaked bead induced the intermediate (zeugopodial) structures between the stump and the graft. The scale bar in B is 2 mm

The skeletal pattern caused by the surgery was investigated. Chick embryos were harvested on day 11 of incubation. The skeletal pattern was visualized by Alcian blue staining. The skeletal pattern of an intact (non-amputated) chick limb is shown in Fig. 2b. Limb bud simply amputated one-quarter from the proximal border caused severe loss of distal parts (Fig. 2c). Importantly, the distal half of the stylopod was always absent and no zeugopodial elements were ever observed. When one-quarter of a distal part and one-quarter of a proximal part were joined and a PBS-soaked bead was placed between them, the zeugopod elements were not reformed. The autopod elements were directly elongated from the stylopod element (Fig. 2d, Table 1). When the Fgf2 + Fgf8-soaked bead was placed on the border of the graft, the zeugopod elements were induced (Fig. 2e, Table 1, Additional file 1: Figure S1). Some exhibited relatively normal radius and ulna cartilage (*n* = 9/21; 43%), and some exhibited truncated but identifiable zeugopodial elements (*n* = 7/21; 43%, Additional file 1: Figure S1). This suggests that ectopic Fgf signaling mediates the intercalary response in chick limb buds.

**Table 1:** Fgf-mediated intercalary responses in st. 24 chicken limb buds

	Zeugopod induced	Intermedial cartilage formed	No zeugopod	Total
FGF2 + FGF8	9	7	5	21
PBS	0	2	18	20

### HoxA11 expression, cell death, and proliferation in the intercalary reaction

To characterize the induced intermediate structure by Fgf2 + Fgf8 application, a molecular marker gene of a zeugopod was necessary. *HoxA11* has been used as a typical zeugopodial marker gene in amniotes. *HoxA11* is first expressed from the very distal tip of a limb bud, including to the presumptive autopodial region. Later, *HoxA11* expression starts being suppressed in a distal tip of a limb bud around st. 25 [32], and the *HoxA11* expression domain is relatively restricted in the zeugopodial domain [32–34]. A knockout of the *Hox11* paralogous group (*Hoxa11*, *Hoxc11*, and *Hoxd11*) results in severe underdevelopment of the zeugopodial elements, and *relatively* normal autopod development [35]. These findings indicate that HoxA11 is a reasonable zeugopodial marker gene.

**Fig. 3:**
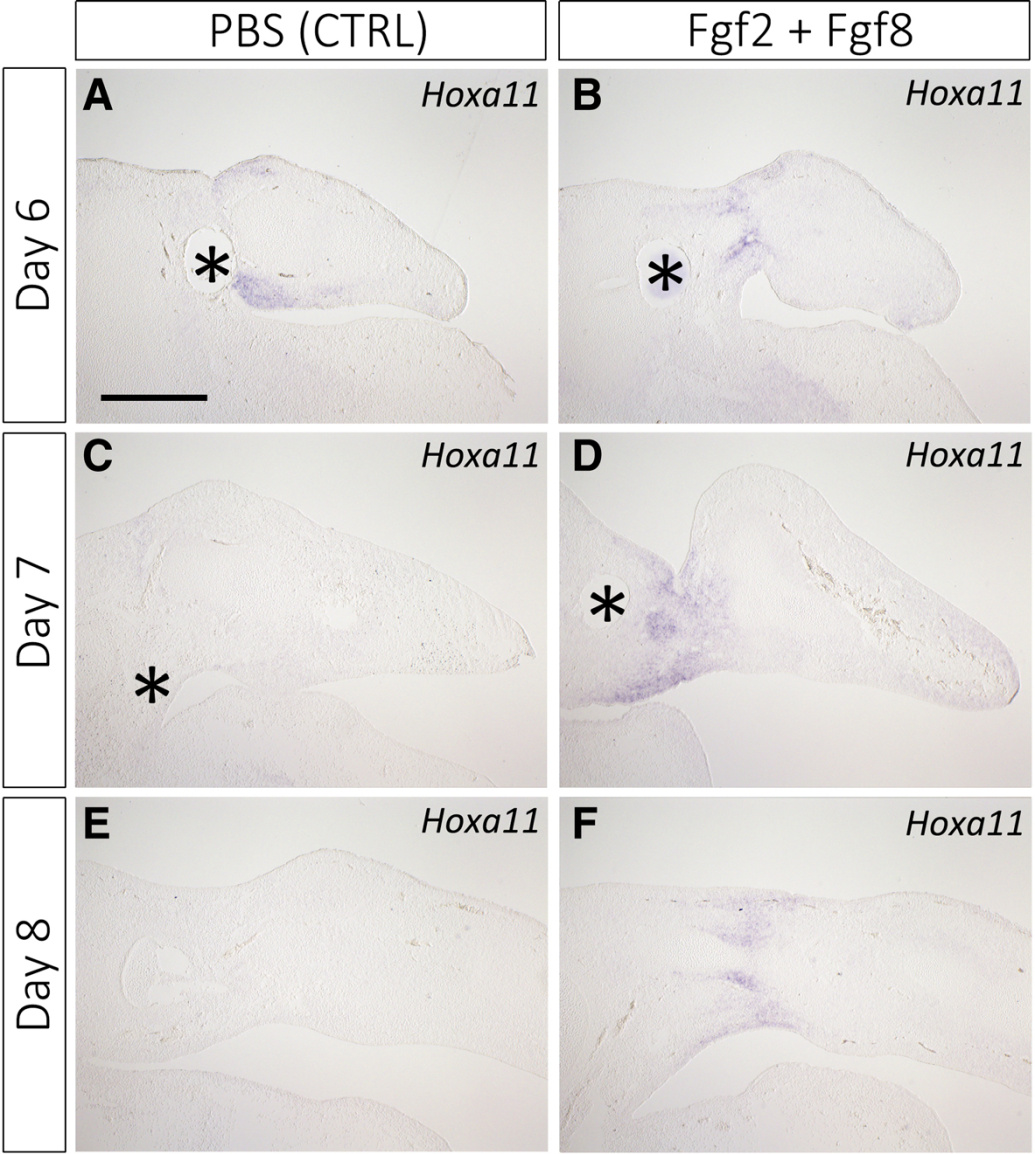
*HoxA11* expression pattern in the operated limbs. *HoxA11* expression in the control (PBS) samples (**a, c, e**) and Fgf2 + Fgf8 bead-grafted limb buds (**b, d, f**). **a**, **b** *HoxA11* expression remained at the distal parts on day 6 (2 days after surgery). *HoxA11* expression disappeared in the control samples at later time points, however (**c, e**). **d**, **f** *HoxA11* expression was observed in the proximal region in the Fgf2 + Fgf8 bead-grafted limbs. Asterisks indicate the grafted beads. The scale bar in A is 500 μm

The skeletal pattern strongly suggested that the zeugopodial elements were induced by ectopic Fgf2 + Fgf8 application (Fig. 2e). To confirm this further, *HoxA11* expression was investigated in the operated limb buds (Fig. 3). Removal of the presumptive zeugopodial part and bead grafting were performed. Samples for in situ hybridization were collected 2–4 days after surgery. In the control, *HoxA11* expression was detectable in the proximal region of the grafted distal part (Fig. 3a). On day 7 (3 days after surgery), *HoxA11* expression disappeared and became unrecognizable (Fig. 3c). In later periods, no *HoxA11* was detectable (Fig. 3e, data not shown). In contrast, *HoxA11* expression was maintained in the Fgf2 + Fgf8 bead-grafted limb (Fig. 3b, d, f). On day 6 (2 days after surgery), *HoxA11*expression was identifiable on the control side (Fig. 3a, b). Notably, *HoxA11* remained detectable within the proximal region in the later stages (Fig. 3d, f). The *HoxA11* expression domain appeared to be induced within the stump region. This result indicates that *HoxA11* expression was induced under the condition of ectopic Fgf signaling and discontinuity of the PD positional values.

**Fig. 4:**
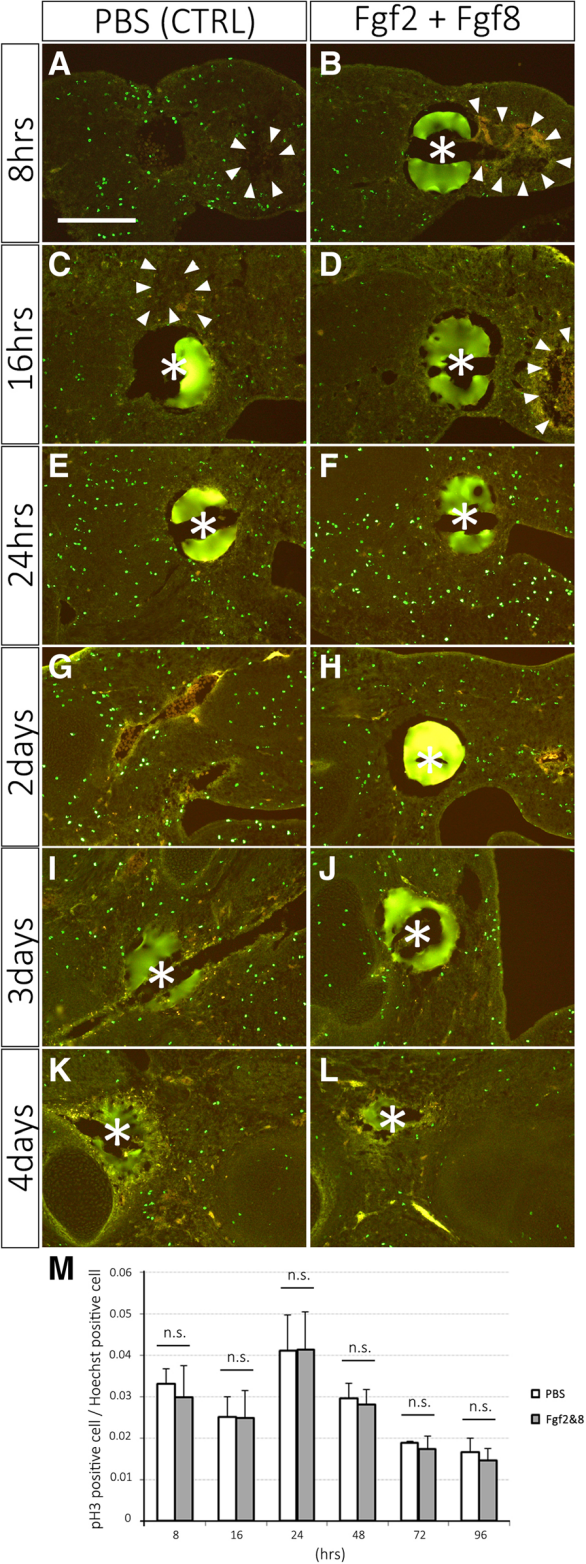
Mitogenic activity was measured by pH 3 immunofluorescence. The left column shows the control samples with the PBS-soaked bead. The right column shows the limb buds with the Fgf2 + Fgf8-soaked bead. Asterisks and arrowheads indicate the beads and the ischemic region, respectively. The scale bar in A is 250 μm. (M) The summary of the result of A–L and the statistical analysis. n.s. = no significant difference

**Fig. 5:**
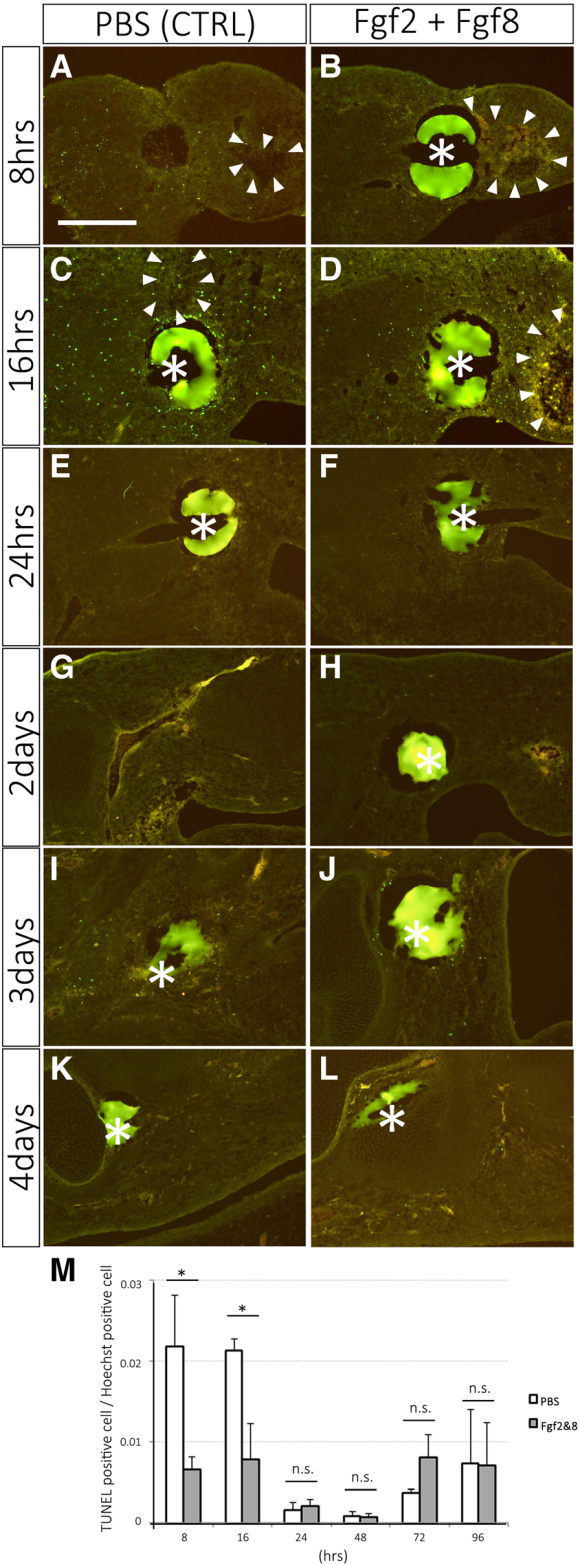
Apoptosis was investigated by TUNEL analysis. The left column shows the control samples with the PBS-soaked bead. The right column shows the limb buds with the Fgf2 + Fgf8-soaked bead. Asterisks and arrowheads indicate the beads and the ischemic region, respectively. The scale bar in A is 250 μm. (M) The summary of the result of A–L and the statistical analysis. n.s. = no significant difference.* = *P* < 0.05

Cell proliferation and apoptosis were also investigated in the intercalary response (Figs. 4, 5). Mitogenic activity was measured by phosphorylated histone H3 (pH 3), which is used as a specific marker of S-phase. The pH 3 positive cells were counted from three independent samples at each time point (Fig. 4m). Cell counting was performed around the place where bead was observed. Unexpectedly, no apparent difference between the control and the Fgf-grafted limb was observed at any time point (Fig. 4m). At the early time points (8 and 16 h), however, the ischemic region, where pH 3 positive cells were not detected, was sometimes observed (Fig. 4a–d, arrowheads). Apoptotic cells were visualized by TUNEL analysis (Fig. 5). Apoptotic cells became apparent within 16 h (Fig. 5a–d, m). Fgf2 + Fgf8 application appeared to suppress apoptosis compared to the control (Fig. 5a–d, m). At later time points, few apoptotic cells could be detected in either sample (Fig. 5e–l). These results suggest that neither cell proliferation nor cell death is the primary force of the intercalation response in chick limb buds.

### Lineage tracing analysis reveals proximal dominant contribution

**Fig. 6:**
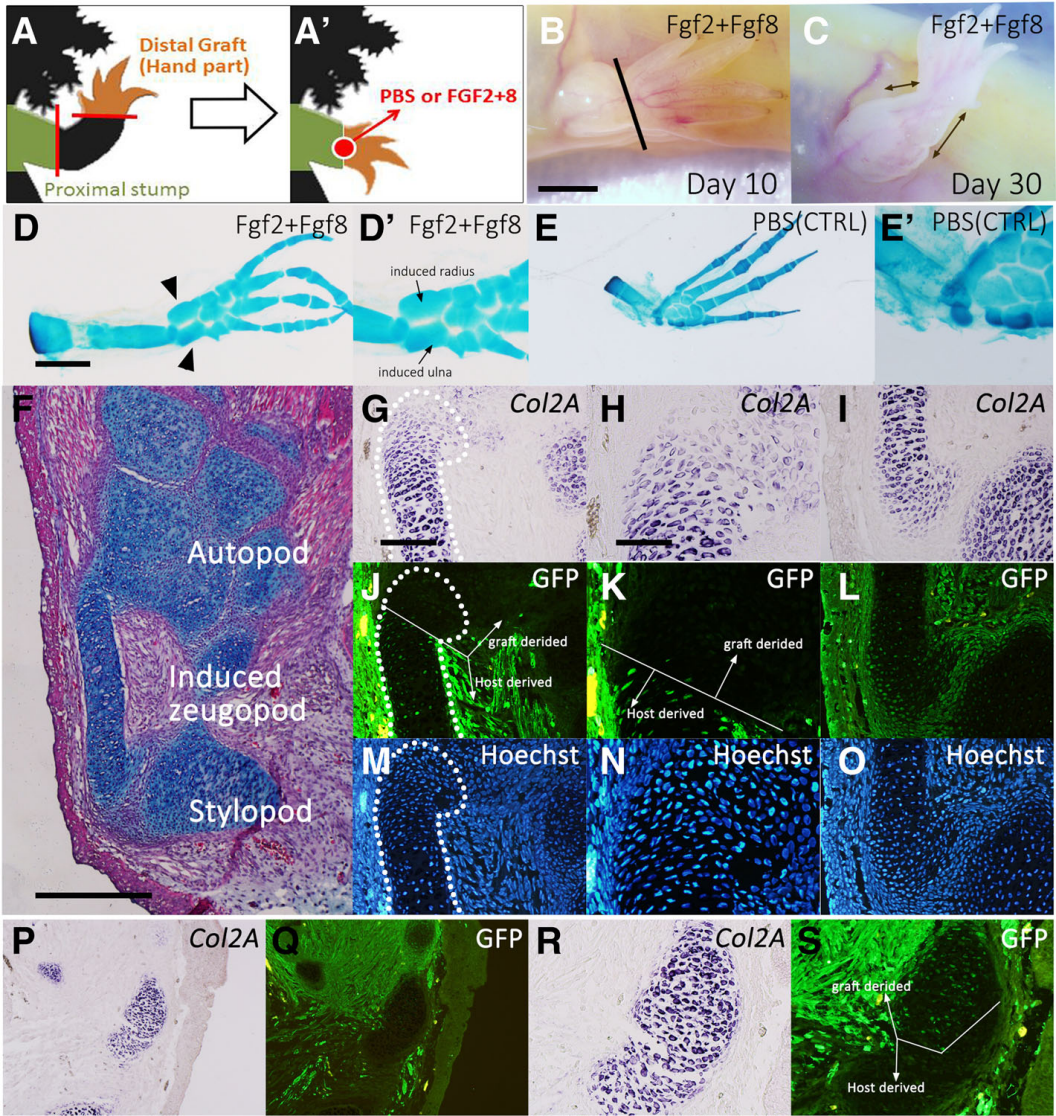
Re-evaluation of cellular contribution in the axolotl intercalary response. **a** Schematic diagram of the experimental design. **b** Hand grafting 10 days after surgery. The line indicates the approximate border of the graft. The scale bar is 3 mm. **c** Day 30. The elongated portion is recognizable between the grafted hand and the stump (double-headed arrows). **d**, **e** Skeletal pattern was revealed by Alcian blue staining. **d** Fgf2 + Fgf8 bead-grafted sample. Arrowheads indicate the two induced cartilages between the graft and the stump. (D’) Higher magnification view of D. **e** PBS-soaked bead grafting resulted in no intercalary responses (E’). **f** Histological observation of the Fgf2 + Fgf8 bead-grafted limb. Cartilage formation is readily evident between the hand graft and the stump. The scale bar indicates 1 mm. **g**–**o** The identical section underwent in situ hybridization and immunofluorescence. (G–I) *Type II collagen* (*Col2A*) expression pattern in the intercalary induced cartilage. (J–L) GFP expression pattern in the intercalary induced cartilage. The host was a GFP transgenic axolotl. Proximally derived cells were GFP-positive. **m**–**o** Hoechst staining for nuclei. **g**, **j**, and **m** are the distal regions of the induced zeugopod, and **i**, **l**, and **o** are the proximal. H, K, and N are the higher magnification views of **g**, **j**, and **m**, respectively. The scale bars in G and H are 500 and 200 μm, respectively. **p**–**s** The same experiment was performed with a normal host animal and a graft derived from a GFP transgenic axolotl. **p**, **r**) *Type II collagen* (*Col2A*) expression pattern in the intercalary induced cartilage. **q**, **s** GFP expression pattern in the intercalary induced cartilage. R and S are higher magnification views

Intercalation in limb regeneration processes has mainly been studied in amphibians. Classic cell lineage analysis revealed that stump tissues contribute to newly induced intermediate structures [36–38]. When a distal (autopodial) part was placed onto an amputated stylopod, the regenerated intermediate (zeugopod) structures consist of stylopod-derived cells. This finding has not, however, been re-evaluated using recent molecular technology. Recently, Fgf-mediated intercalation was demonstrated in axolotl limbs [7, 21]. The hand grafting technique in a mature axolotl was first pioneered by Bryant and Item [39]. The hand part was dissected out and placed onto an amputated stylopod. Essentially, no zeugopodial elements were regenerated ([21]; Fig. 6e). A previous study by our group demonstrated that Fgf2 application between the hand part and the amputated stylopod could induce zeugopodial elements [21]. We first re-examined this using a GFP transgenic and a normal axolotl. The summary of the experimental design is illustrated in Fig. 6a. The hand part was derived from a wild-type axolotl and the host was a GFP transgenic animal. Fgf2 + Fgf8 was used in order to achieve similar experimental conditions to those of the chick experiments. As reported previously, ectopic Fgf application to the border of the graft successfully induced zeugopodial elements (*n* = 6/10; [21]; Fig. 6b–d). No obvious morphological change was observed 10 days after Fgf2 + Fgf8 application (Fig. 6b). A recognizable extension was observed between the stump and the grafted hand part 30 days after the surgery (Fig. 6c). It was difficult to observe for much longer than this because of immuno-rejection of the graft. Alcian blue staining revealed the skeletal pattern (Fig. 6d, e). In the Fgf2 + Fgf8-grafted samples, the induced zeugopodial elements could be observed (Fig. 6d, D’). In contrast, the PBS-soaked bead grafting resulted in no regeneration of zeugopodial elements (Fig. 6e, E’). To investigate the cellular contribution, the Fgf2 + Fgf8 bead-grafted samples were fixed and sectioned (Fig. 6f). Consistently, the histological analysis revealed that the zeugopodial elements were induced by Fgf2 + Fgf8 application (Fig. 6f). The induced cartilaginous elements existed independently from the autopodial and stylopodial skeletal elements (Fig. 6f). The border cells of the induced zeugopod cartilage expressed *Gdf5* (Additional file 2: Figure S2). This suggests that each element was segregated by joint-like formation. Cartilaginous cells were visualized by *type II collagen* (*Col2A)* in situ hybridization, and GFP-positive cells were visualized by immunofluorescence (Fig. 6g–s). For the induced zeugopod, the distal region consisted of two populations, GFP-positive and GFP-negative (Fig. 6j, k, m, n). In contrast, the proximal region of the induced zeugopod was composed of host-derived cells (GFP-positive; Fig. 6l, o). This result was further confirmed in animals that had a GFP-positive graft and a GFP-negative host (Fig. 6p–s). In these animals, the induced zeugopodial elements consist of GFP-positive cells in the distal part and GFP-negative cells in the proximal part (Fig. 6p–s). These results clearly demand renewal of the classic understanding of the intercalary response in amphibian limb regeneration. Consistently, most of the intercalary induced tissues were derived from the proximal tissues. However, distal tissues can also contribute to the intercalary induced tissues in the distal region.

**Fig. 7:**
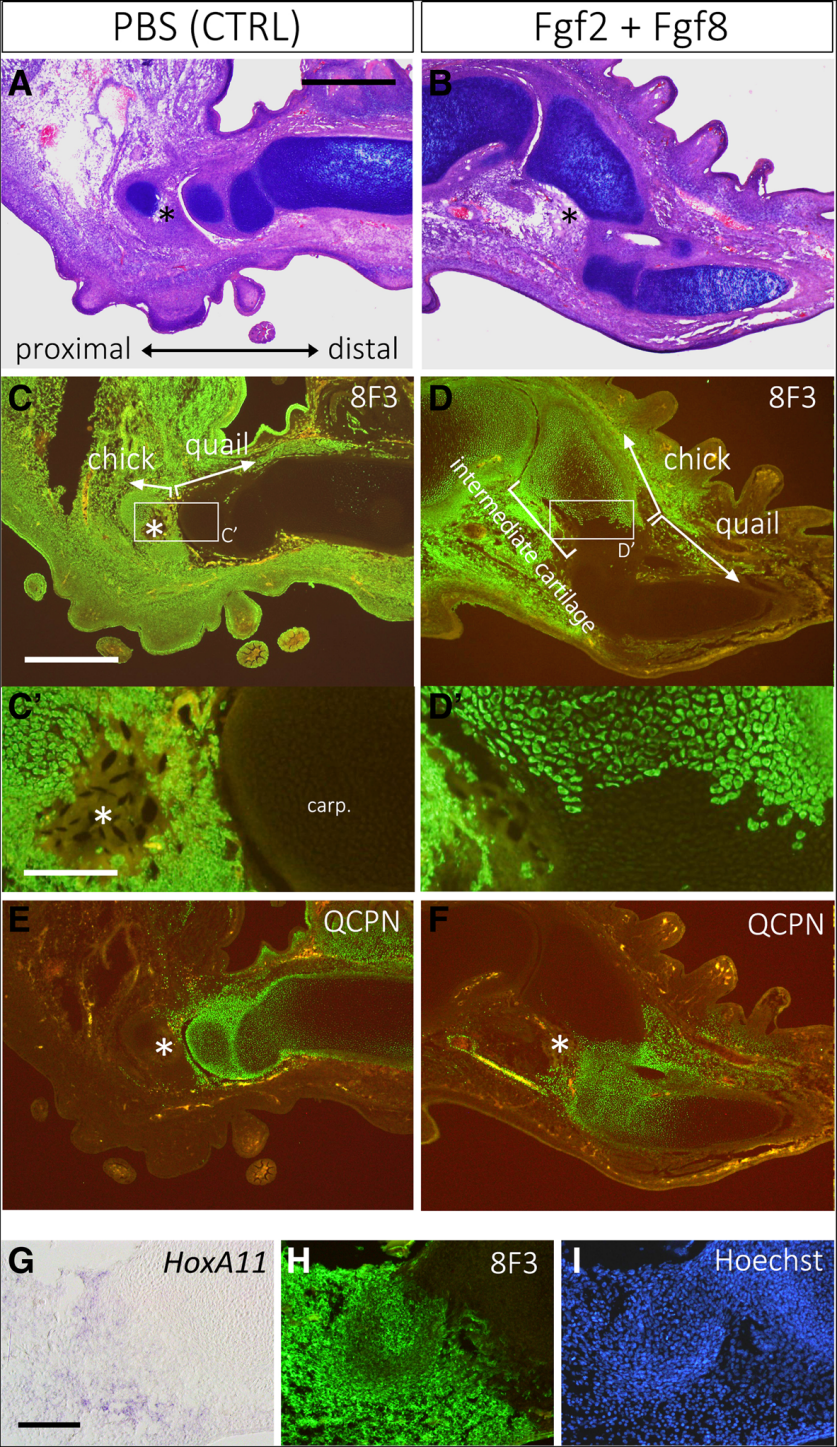
Cellular contribution of the intercalary induced cartilage in chick limb buds. The grafted hand part was derived from a quail limb bud. **a**, **c**, **e** The control sample. **b**, **d**, **f** Fgf2 + Fgf8 bead-grafted limb. **a**, **b** Histological observation. (**c–f**) Immunofluorescent analysis using the antibody specific for chick cells (8F3; **c**, **d**) and for quail cells (QCPN; **e**, **f**). C’ and D’ are the higher magnification views of the boxed regions in **c** and **d**, respectively. Asterisks indicate the beads. Carp. = carpal cartilage. (G–I) The relationship between the *HoxA11* expression domain and the 8F3 expression domain was investigated in the adjacent sections. **g** *HoxA11* expression. **h** 8F3 antigen expression on the adjacent section. I is Hoechst staining for nuclei and is the identical section to H. The scale bars in A, C, C’, and G are 500, 500, 100, and 100 μm, respectively

As shown above, the classical understanding of the cellular contribution of intercalary regenerated tissue needs to be revised. Based on the results shown in Fig. 6, the cellular contribution of chick intercalary induced tissues was investigated (Fig. 7). Quail-chick chimeras have been used for cell lineage tracing experiments in chick developmental studies. A distal limb bud was obtained from a quail limb bud, and a chicken limb bud was used as a host. Samples were fixed on day 7 after surgery. Histological observation revealed that Fgf application induced the cartilage structure between the distal graft and the stump whereas the PBS-soaked bead grafting did not give rise to any ectopic structures (Fig. 7a, b). To determine the cell contribution, chick-specific antigen (8F3) was used in the immunofluorescence (Fig. 7c, d). In the control, a clear boundary could be observed (Fig. 7c, C’). There was no cartilage consisting of both chick-derived and quail-derived cells (Fig. 7c, C’). It was clear that the host cells penetrated into the distal region (Fig. 7c, d). In limb development, migrating cells, such as muscle cells, endothelial cells, fibroblasts, and neural cells, toward a distal region exist, which accounts for the 8F3-positivecells in the distal region (Fig. 7c, d). The Fgf2 + Fgf8-induced cartilage constituted of the two populations (Fig. 7d). Within the stylopod and the autopod region, cartilage consisted of either population (Fig. 7d). It was evident that two populations contributed to the intercalary induced cartilage (Fig. 7d’). Within the induced cartilage, most of the cartilage cells were derived from the host (8F3-positive). The distal region of the induced cartilage was derived from the graft (8F3-negative; Fig. 7D’). QCPN immunofluorescence was conducted at the location of the graft (quail) cells (Fig. 7e, f). The results were perfectly consistent with 8F3 immunofluorescence. The cellular contribution was similar to that of intercalary regeneration in an axolotl (Fig. 6). This result suggests that intercalation in chick limb buds is regulated in a similar manner as that of urodele amphibians.

We also investigated the relationship between *HoxA11* re-expression and cellular contribution. In this experiment, the host was derived from a chick embryo and the graft was from a quail limb bud. Samples were fixed 4 days after the surgery. *HoxA11* expression was determined by in situ hybridization (Fig. 7g), and chicken cells were clarified by 8F3 immunofluorescence on the adjacent section (Fig. 7H). The *HoxA11* expression domain appeared to be within the 8F3-positive domain (Fig. 7g–i). This suggests that the proximal cell population mainly generates *HoxA11*-positive zeugopodial elements during the intercalary response.

## Discussion

### Intercalary regulation in chick limbs

Intercalary regulation is quite common in amphibian limb regeneration. Bryant and her colleagues first proposed that distal and proximal interaction creates intermediate structures [4]. This concept has not, however, been applied to chick limbs [40]. A classical grafting experiment reported that intercalation did not take place if a distal part was transplanted onto an amputated limb bud at an expected stylopod [14]. It is of note that the induction of intermediate structures in this grafting experiment was reported in chick limb buds in the early stage [41, 42]. Very early chicken limb buds, especially around st. 20, do not have a distinguished zeugopod-specified population [43, 44]. The very distal tip of an early-stage limb bud contains expected autopods and zeugopods. *HoxA13*, an autopod marker gene, is upregulated from st. 21 [32, 45]. Therefore, it is not surprising that the distal graft contains zeugopodial parts in these early experiments. As shown in Fig. 1, a limb bud at st. 24 has distinguished autopod, zeugopod, and stylopod regions. Furthermore, zeugopodial cartilaginous elements were already specified, and recognized as *Pg-h*-positive radius/ulna cartilage (Fig. 1f). The use of st. 24 limb buds decreased the chance to contaminate any zeugopodially specified cells in the grafted distal part (Fig. 1b). Nevertheless, it remains possible that a distal part was involved in a zeugopodial part. TUNEL analysis revealed that Fgf2 + Fgf8-soaked bead grafting suppressed apoptosis 16 h after the surgery (Fig. 5c). It has already been demonstrated that Fgf signaling has the ability to suppress cell death in chicken limb buds [46, 47]. This apoptosis pattern implies that contaminated zeugopodial cells were eliminated without exogenous Fgf application, and that exogenous Fgf application allowed these populations to survive. We cannot dismiss this possibility completely. Cell lineage analysis, however, revealed that most of the induced zeugopodial elements were derived from the proximal stump tissues (Fig. 7). Thus, even if zeugopodial cell contamination were present, its contribution to the intercalary induced structure must not be great. As for the proximal part (the stump), the skeletal pattern obtained in the grafting experiments (Fig. 2c, d, Additional file 1: Figure S1) indicates that no zeugopodial elements are contained. Many samples showed that the induced zeugopodial elements directly extended from the proximal half of the stylopod (Additional file 1: Figure S1). This skeletal pattern strongly suggests that the amputation was correctly performed at the middle of the stylopod, at least in those samples. Given these findings, it is evident that chick limb buds, like urodele amphibians, have the ability to induce the intercalary response.

### Proximal-dominant cellular contribution to induced cartilage

Classical observation showed that intercalary induced tissues were only derived from proximal tissues [36–38]. The present study may demand a small modification of this insight. The labeling technology employed in classical studies was not as accurate compared to that of present-day technology [36–38, 48]. The present study used a GFP transgenic animal to trace cell lineage. Furthermore, a hand graft was taken instead of a blastema graft in the present manuscript. Compared to a blastema, a mature hand was obvious about an amputation site since carpal cartilages instruct a guaranteed amputation site. Because of this, the elimination of zeugopodial elements in the hand grafting experiment was certain. Therefore, it is thought that the combination of the GFP transgenic animal and the hand grafting surgery resulted in a more reliable experimental design. Consistent with the results of classical experiments, it was clearly demonstrated that proximal tissues are the major contributor of intercalary induced tissues (Fig. 6). Detailed cell lineage analysis also revealed that the distal part of the intercalary induced tissues was derived from the distal tissues (Fig. 6g–s). This distal cell contribution to proximal regenerating tissues was already predicted in our previous studies [7, 49]. Similar cellular contribution in an intercalary response was reported in a planarian study [50]. If a small piece of head piece is grafted into a tail piece, an intercalary response can be induced that results in restoration of the pharyngeal region (intermediate). The induced structure consists mainly of host cells from the tail piece although there are some from the head piece. Given this, coordinated cellular contribution is likely a conserved mechanism of the intercalary response from invertebrates to vertebrates.

Cell lineage tracing analysis in chick intercalary responses revealed the proximal-dominant contribution to intercalary induced tissues (Fig. 7). This is consistent with the axolotl results (Fig. 6). The distal part of the intercalary induced zeugopod was derived from the distal graft although the proximal part was derived from the proximal stump tissues (Fig. 7d, f). Importantly, *HoxA11* expression was re-induced in the stump (Fig. 7g–i). Generally, proximal parts consist of much more differentiated and determined cells compared to distal parts. Whether or not determined stylopod cells are reprogrammed into zeugopodial cells remains to be investigated. Given the reprogramming of the stylopod tissues, the application of an Fgf2 + Fgf8 bead may play a part. Application of ectopic Fgf2 + Fgf8 in a skin wound is sufficient to induce a blastema in urodele amphibians [18]. Furthermore, blastema induction by Fgf signaling can be observed in some species [17, 51]. In addition, blastemas always possess distal identities [52]. Fgf2 + Fgf8 application can actually induce a blastema with distal information from a proximal structure. Similarly, Fgf2 + Fgf8 may induce cells with a distal identity from proximal tissues in chick limb buds. Although the detailed mechanisms remain largely unknown, it is very likely that cells in the proximal region are transformed into relatively distal cells. This process to generate distal cells from proximal cells may be called “regeneration” since limb regeneration basically involves the generation of distal structures from remaining proximal stump tissues.

### Conserved molecular mechanism of the intercalary response

Fgf signaling is the key molecular signaling that regulates the intercalary response. This was first speculated by Shimizu-Nishikawa et al. in *Xenopus* limb development [22]. Basically, *Xenopus* limb buds lack intercalary ability. The ectopic application of Fgf8, however, induced intercalary responses [22]. The involvement of Fgf signaling in the intercalary response in a urodele amphibian was subsequently reported [21]. Fgf2 application between a hand and a stylopod could induce the zeugopodial elements [21]. This observation was confirmed in the present study (Fig. 6). Even in mice, Fgf signaling regulates intercalary limb developmental mechanisms [20]. Multiple knockout of Fgf genes resulted in a loss of intermediate skeletal elements although the autopod and stylopod were relatively normal. In the case of invertebrates, intercalary regeneration is well known in planarian regeneration [1]. It is still uncertain if Fgf signaling mediates the intercalary regeneration in planarians. An Fgf receptor-like gene, *nou-darake*, was, however, reported in a planarian [53]. Knockdown of *nou-darake* resulted in misformation of the proximodistal structure. Furthermore, it was reported that mitogen-activated protein kinase (MAPK)/extracellular signal-related kinase (ERK) signaling is essential for intercalary planarian regeneration [54]. ERK is well recognized as one of the major targets of Fgf signaling [55]. Disruption of ERK signaling impairs planarian regeneration. Thus, it is likely that an Fgf-mediated intercalary mechanism plays a role in both limb morphogenesis and in more general morphogenesis.

The present study shows that the Fgf signaling-dependent intercalary response can be withdrawn in a chick limb bud. It is reasonable to consider that chick limb development is regulated in a similar manner as mouse limb development. Integrity between the present study and classic studies is found when we focus on the *Fgf* expression domain in a chick limb bud. *Fgf* genes are expressed in the apical ectodermal ridge (AER), which is located at the very distal end of a developing limb bud [56, 57]. A narrow distal domain in a limb bud mesenchyme expresses Fgf10 to maintain *Fgf* genes in the AER. This suggests that the expected zeugopodial region is Fgf-free as the developmental stages progress. In the very early stages, limb buds are very proximodistally thin. Therefore, Fgfs from the AER and mesenchyme may be able to reach a proximal domain. This may explain the intercalary response in the early stages of chick limb buds [41, 42]. In later stages, limb buds become longer. A distal graft cannot provide sufficient Fgfs to proximal tissues from the distal tip because of its own width. This may explain why the intercalary response could not be observed in relatively later stages. This hypothesis fits with the previous studies that demonstrated short range signal from the AER induces the intercalary response in early chicken embryo [58–61]. Interestingly, *Fgf8*, which is expressed in the AER in chick limb buds, is expressed in axolotl limb blastema “mesenchyme” [62, 63]. Furthermore, *Xenopus Fgf8* is expressed in a blastema epithelium [64]. Application of an ectopic Fgf8 into a *Xenopus* limb bud mesenchyme causes intercalary regulation [22]. This appears to be a reasonable relationship between an *Fgf* expression domain and an intercalary ability. Evolutionary transition of AER expressing an Fgf gene expression domain would account for the evolutionary loss of the intercalary regeneration system. Regardless, chick limb bud cells continue to retain reactivity to Fgfs, leading to the intercalary response.

### Regeneration in amniotes

The present results can be regarded as regeneration of intermediate structures. To date, the induction of a regeneration blastema, which is a structure with a distal positional identity, remains a future challenge that needs to be solved in amniotes. There are many cases, however, in which “distal tissues” can be obtained. For example, when a serious tumor grows on a lower arm and demands an amputation, a doctor might obtain a hand part from the abscised limb. It may then be feasible to use the hand part with Fgf application to restore the lost lower arm tissues. Alternatively, extracellular matrix (ECM) from distal tissues might be utilized instead of the hand graft. Recently, it was shown that ECM carries positional information. We may use these distal tissues to induce an intercalary regeneration response.

We still do not how to induce a blastema in amniotes. The organ-level regeneration inducers in amphibians have already been reported, however [51]. Given the conservation of the Fgf signaling-based intercalary mechanism from amphibians to amniotes, it is not surprising that the same inducers play a role in blastema induction in amniotes. Urodele amphibians may not be such unique organisms, and we may be on the way to a better understanding of how to mimic some of their special abilities.

## Conclusion

Generally, amniotes do not have organ-level regeneration ability. This may be attributable to loss of a genetic mechanism regulating regeneration ability. In the present study, we demonstrated that the intercalary responses, which are used in limb regeneration in regeneration competent animals, could be induced by the exogenous Fgf2 and Fgf8 application in chicken limb buds. From this point of view, amniotes, at least chick embryos, may maintain a part of the regeneration mechanism. All of our experiments were performed in the embryonic period. Although the reason why the intercalary responses cannot be seen in a postembryonic body remains unknown, our findings imply that the common organ-level regeneration inducers can induce regeneration responses in amniotes.

Intercalation in regeneration involves interaction of cells having different positional identities. When cells recognize each other as non-neighbor cells, those cells are generating intermediate cells until they acquire neighbor cells. Fgf-signaling influences on cells to recognize and distinguish neighbor cells in the chick limb buds and axolotl limbs. Positional identity has remained an unresolved issue in biology. The results of the present study may contribute to the understanding of positional identities and recognition in limb development and regeneration.

## Additional files

Additional file 1: **Figure S1.** Skeletal pattern of the intercalary regenerated chick wing by Fgf2 + Fgf8 bead grafting. Skeletal pattern was visualized by Alcian blue staining. The scale bar is 2 mm. (JPEG 1535 kb)

Additional file 2: **Figure S2.** Joint-like formation between the host stylopod and the induced zeugopod by Fgf2 + Fgf8 application. (A) *Gdf5* expression. (B) *Type II collagen* expression. The scale bar is 2 mm. (JPEG 2568 kb)
