# Supplementary figures and images for: Ectopic Fgf signaling induces the intercalary response in developing chicken limb buds
Source: Zoological Lett. 2018 Apr 19;4:8. doi: 10.1186/s40851-018-0090-2 (PMC5907462; doi:10.1186/s40851-018-0090-2)

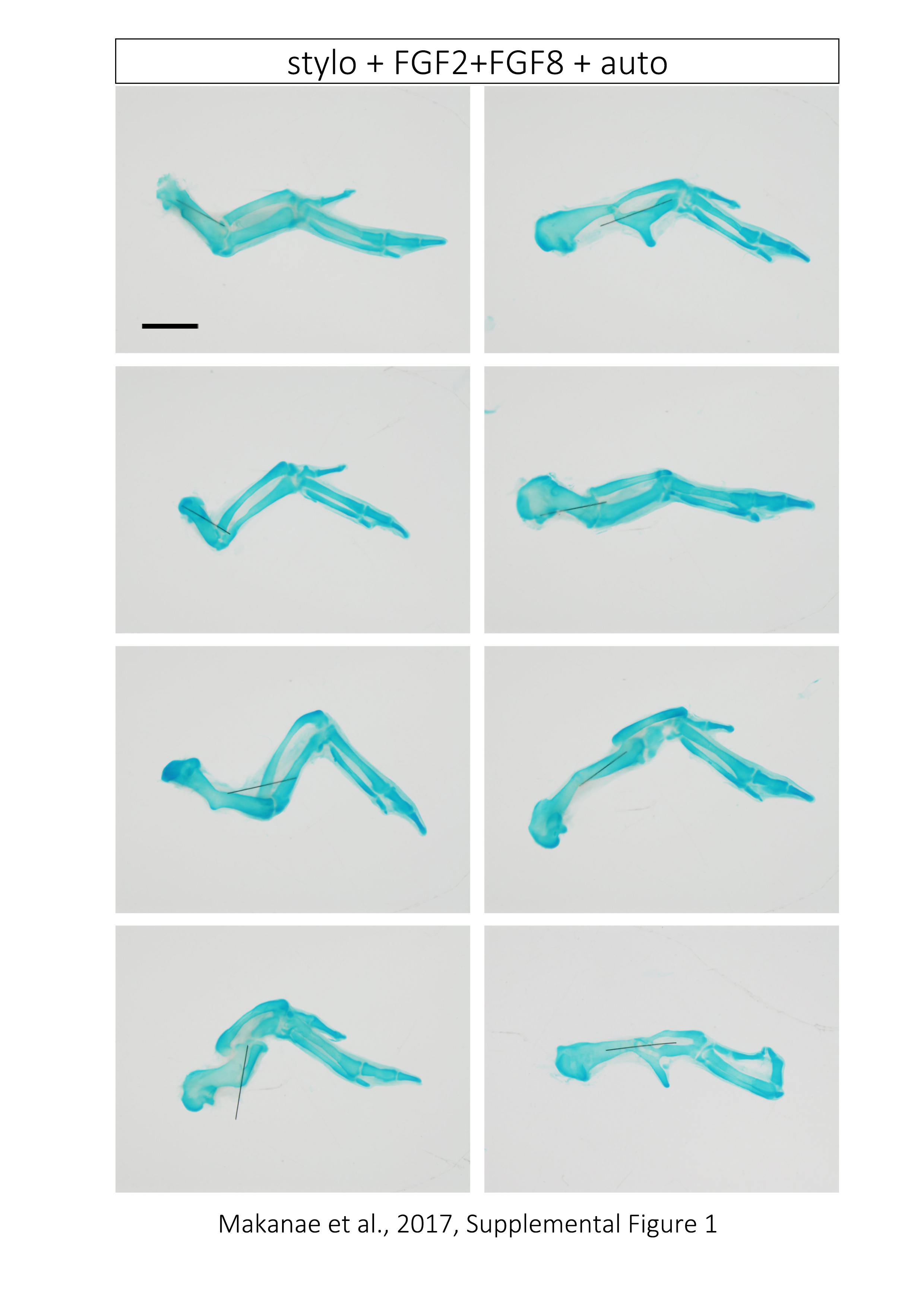

Supplement: Supplementary file 1 — Figure S1. Skeletal pattern of the intercalary regenerated chick wing by Fgf2 + Fgf8 bead grafting. Skeletal pattern was visualized by Alcian blue staining. The scale bar is 2 mm. (JPEG 1535 kb) [file 40851_2018_90_MOESM1_ESM.jpg]

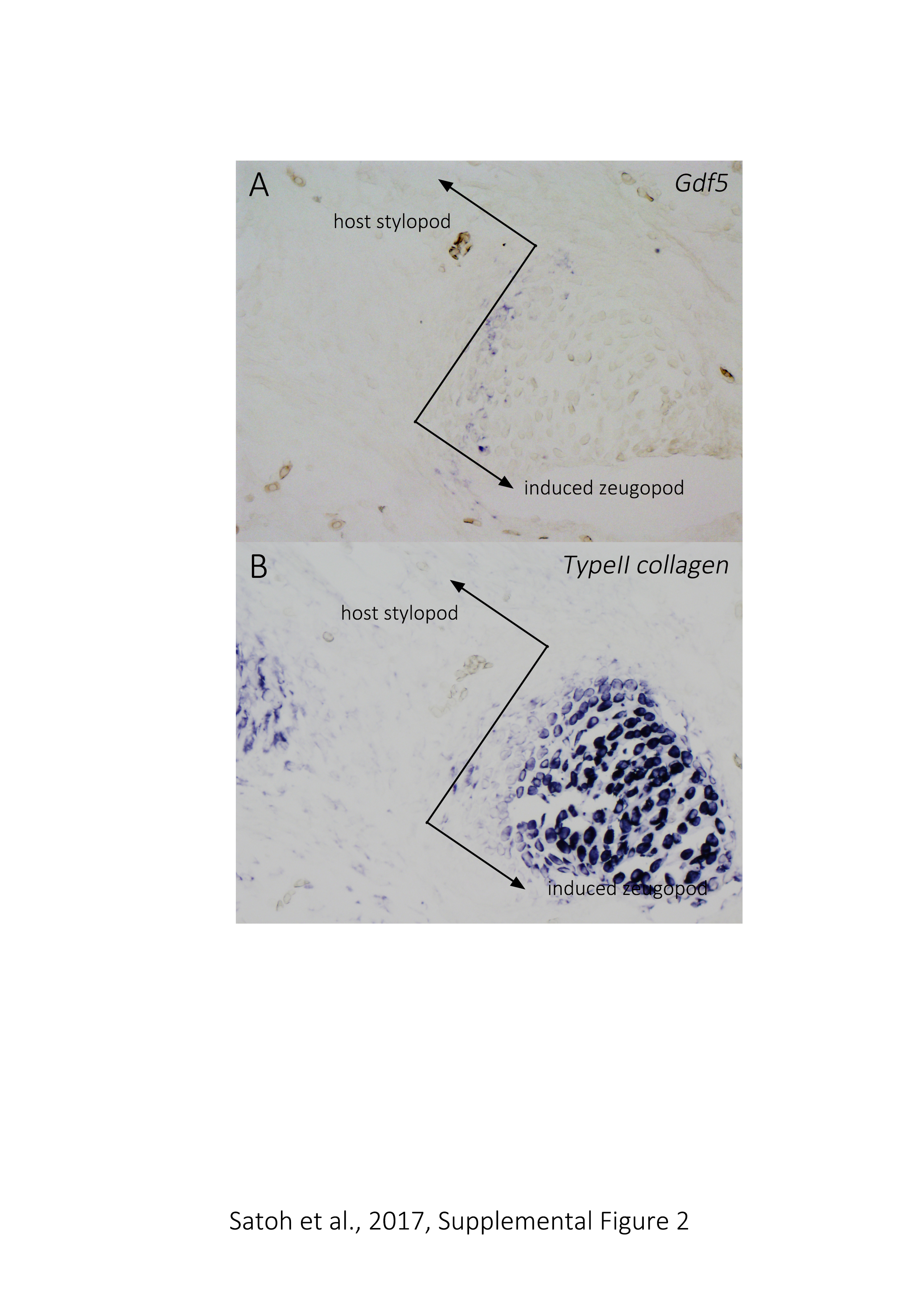

Supplement: Supplementary file 2 — Figure S2. Joint-like formation between the host stylopod and the induced zeugopod by Fgf2 + Fgf8 application. (A) Gdf5 expression. (B) Type II collagen expression. The scale bar is 2 mm. (JPEG 2568 kb) [file 40851_2018_90_MOESM2_ESM.jpg]
